# Reservoirs of antimicrobial resistance genes in retail raw milk

**DOI:** 10.1186/s40168-020-00861-6

**Published:** 2020-06-26

**Authors:** Jinxin Liu, Yuanting Zhu, Michele Jay-Russell, Danielle G. Lemay, David A. Mills

**Affiliations:** 1 Department of Food Science and Technology, Robert Mondavi Institute for Wine and Food Science, University of California, Davis, One Shields Ave, Davis, CA 95616 USA; 2 Foods for Health Institute, University of California, Davis, One Shields Ave, Davis, CA 95616 USA; 3 Western Center for Food Safety, University of California, Davis, Davis, CA 95616 USA; 4 USDA ARS Western Human Nutrition Research Center, 430 West Health Sciences Dr, Davis, CA 95616 USA; 5 Genome Center, University of California, 451 Health Sciences Dr., Davis, California, 95616 USA; 6 Department of Nutrition, University of California, Davis, One Shields Ave., Davis, CA 95616 USA; 7 Department of Viticulture and Enology, Robert Mondavi Institute for Wine and Food Science, University of California, Davis, One Shields Ave, Davis, CA 95616 USA

**Keywords:** Raw milk, Antimicrobial resistance, Metagenomics, Public health

## Abstract

**Background:**

It has been estimated that at least 3% of the USA population consumes unpasteurized (raw) milk from animal sources, and the demand to legalize raw milk sales continues to increase. However, consumption of raw milk can cause foodborne illness and be a source of bacteria containing transferrable antimicrobial resistance genes (ARGs). To obtain a comprehensive understanding of the microbiome and antibiotic resistome in both raw and processed milk, we systematically analyzed 2034 retail milk samples including unpasteurized milk and pasteurized milk via vat pasteurization, high-temperature-short-time pasteurization, and ultra-pasteurization from the United States using complementary culture-based, 16S rRNA gene, and metagenomic sequencing techniques.

**Results:**

Raw milk samples had the highest prevalence of viable bacteria which were measured as all aerobic bacteria, coliform, and *Escherichia coli* counts, and their microbiota was distinct from other types of milk. 16S rRNA gene sequencing revealed that *Pseudomonadaceae* dominated raw milk with limited levels of lactic acid bacteria. Among all milk samples, the microbiota remained stable with constant bacterial populations when stored at 4 °C. In contrast, storage at room temperature dramatically enriched the bacterial populations present in raw milk samples and, in parallel, significantly increased the richness and abundance of ARGs. Metagenomic sequencing indicated raw milk possessed dramatically more ARGs than pasteurized milk, and a conjugation assay documented the active transfer of *bla*_CMY-2_, one ceftazidime resistance gene present in raw milk-borne *E. coli*, across bacterial species. The room temperature-enriched resistome differed in raw milk from distinct geographic locations, a difference likely associated with regionally distinct milk microbiota.

**Conclusion:**

Despite advertised “probiotic” effects, our results indicate that raw milk microbiota has minimal lactic acid bacteria. In addition, retail raw milk serves as a reservoir of ARGs, populations of which are readily amplified by spontaneous fermentation. There is an increased need to understand potential food safety risks from improper transportation and storage of raw milk with regard to ARGs.

Video Abstract

## Background

Unpasteurized milk for human consumption is currently legalized for sale in 30 states either in retail stores (*n* = 13) or at local farms (*n* = 17) [1] in the USA, and the demand for raw milk is increasing [2, 3]. Despite the proposed health benefits of raw milk for humans [4–6], contamination of raw milk with zoonotic pathogens including *Campylobacter* spp., Shiga toxin–producing *Escherichia coli*, and *Salmonella enterica* have been well documented leading to serious illnesses [7–11]. In addition to the presence of potential pathogenic bacteria, raw milk contains antibiotic-resistant microbes [12–16], and thus the incorporation of raw milk into daily diet may facilitate the dissemination of antimicrobial resistance genes (ARGs) to the human gastrointestinal tract. At present, a comprehensive understanding of the antibiotic resistome in raw milk is lacking.

The raw milk microbiota has been documented in several studies, but has mostly focused on the milk at farms or during transportation [17–19]. However, in-depth investigations employing high throughput sequencing to examine the microbiota of raw milk at retail stores remain limited. In addition, there is little information on the extent to which the milk microbiota responds to various levels of pasteurization and processing. Raw fluid milk is typically directly consumed; however, it is sometimes deliberately left at room temperature (RT) for 1-5 days to make a product termed clabber [20]. Currently, the production of this “naturally fermented” milk is increasing with the public’s interest in traditional foods [21, 22]. Therefore, it is imperative to understand the dynamic changes of bacterial load, microbiota composition, and resistome content of raw milk during such incubations.

To examine this, a total of 2034 retail milk samples were collected from stores in California, Idaho, Arizona, South Carolina, and Maine. Eight milk brands in California representing 4 types of commercial milk processing (raw, vat pasteurized [Vat], high-temperature short time [HTST], ultra-pasteurized [UHT]) were sampled from 8 independent batches over five months. Vat pasteurization is the original method of pasteurization, which heats milk (typically at 145 °F) in a large tank for at least 30 min. HTST pasteurization, the most common method of pasteurization in the USA, requires the milk temperatures to be at least 161 °F for not less than 15 s, followed by rapid cooling. Compared to HTST, UHT pasteurizes milk at an even higher temperature (280 °F) for 2 s and provides extended shelf-life of milk [23]. Collected milk samples were incubated at both 4 °C and 23 °C for up to 24 h, and the live bacterial load and milk microbiota were characterized during this period. Retail raw milk samples were also obtained in other states from 3 independent purchases for microbiota profiling. The milk resistome was characterized via metagenomic sequencing in selected milk samples. Via extensive sampling, culturing, and sequencing, this study expands our understanding of the microbiota composition and antibiotic resistome of retail milks as well as their response to pasteurization, geography, temperature, and spontaneous fermentation. These findings highlight the potential risk for ingestion and transfer of antimicrobial resistance when consuming raw milk.

## Results

### The dynamics of viable bacterial populations in various types of California retail milk during incubations

**Fig. 1:**
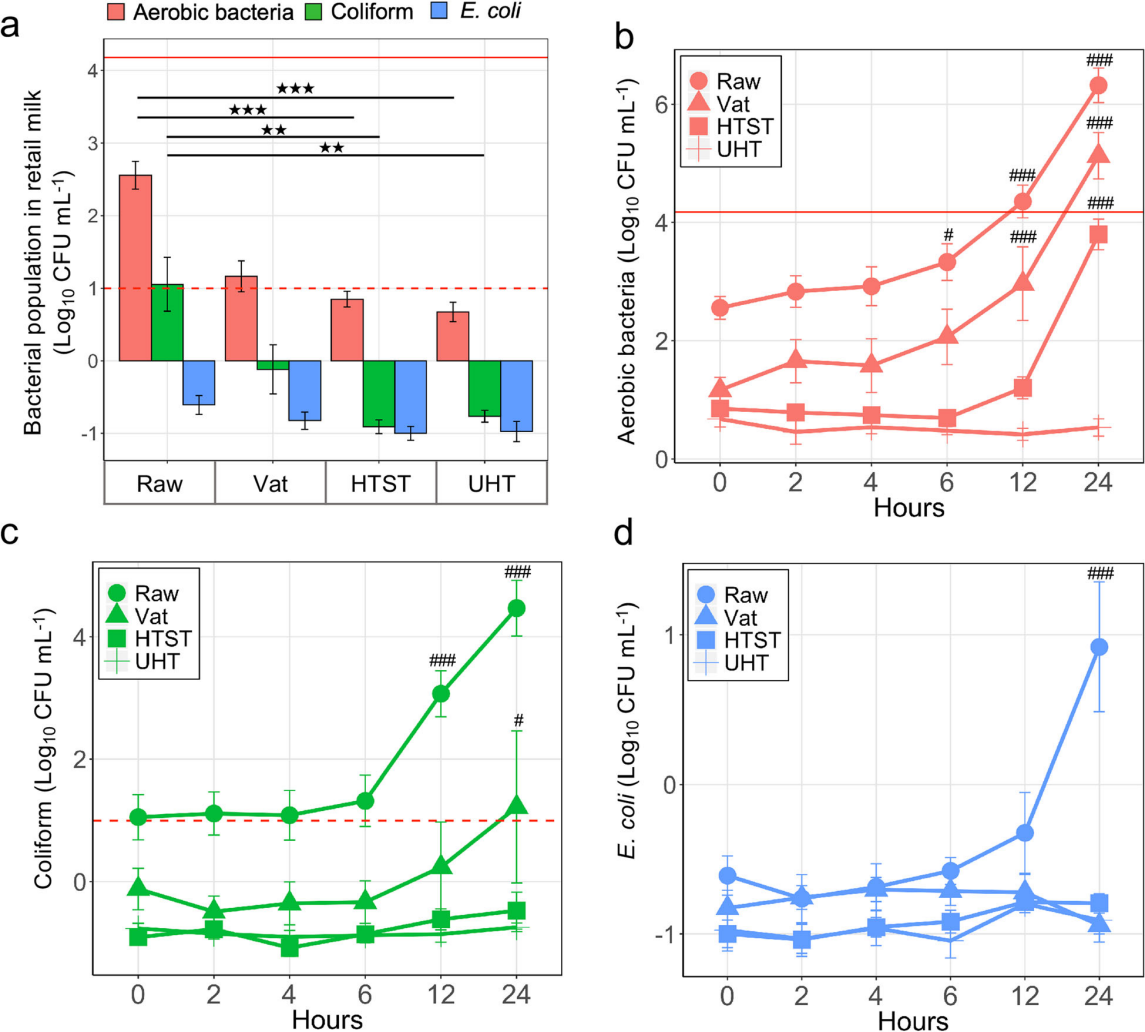
Bacterial population dynamics of retail milk over 24 h room temperature (RT) incubation. **a** Bacterial populations in freshly purchased retail milk (without incubation). **b** Total aerobic bacteria; **c** Coliforms; **d***E. coli;* populations during the RT incubation within 24 h. A total of 1152 milk samples were analyzed; Raw (*n* = 288), HTST (*n* = 432), Vat (*n* = 144), and UHT (*n* = 288). Solid and dashed horizontal lines represent the California milk threshold for aerobic bacteria (< 15,000 bacteria/mL) and coliforms (< 10 coliform/mL), respectively. ^★^*P* < 0.05, ^★★^*P* < 0.01, and ^★★★^*P* < 0.001 for comparison with raw milk of the same bacterial type. ^#^*P* < 0.05, ^##^*P* < 0.01, and ^###^*P* < 0.001 for comparison with start point (0 h) within each type of milk

In order to explore the milk microbiota and antibiotic resistome, it was relevant to first quantify the viable bacteria and understand their dynamic change over incubations across various types of retail milk. In California, retail raw milk samples showed overall higher populations of live bacteria compared to retail milk samples that had been pasteurized. Prior to any incubations, raw milk had the highest absolute abundance of aerobic bacteria (~ 2.56-log), followed by Vat (Dunn test, *P* = 0.14), HTST (*P* < 0.001), and UHT (*P* < 0.001) (Fig. 1a). Coliforms were present in a similar distribution across milk types, with raw milk containing the most bacteria (~ 1.05-log) which was slightly higher than Vat (*P* = 0.36) and significantly more prevalent than HTST and UHT milks (Dunn test, *P* < 0.01 in both cases, Fig. 1a). There was no measurable significant difference in the population of *E. coli* across milk types (Dunn test, *P* > 0.05, Fig. 1a). At all levels, the bacterial population quantified remained stable in a cold environment (4 °C) over 24 h except a drop of *E. coli* at 4 h in raw milk (Supplementary figure 1).

According to The California Department of Food and Agriculture (CDFA), it is unlawful to distribute raw milk which contains more than 15,000 bacteria per milliliter or more than 10 coliform bacteria per milliliter [24]. Given that milk can be unintentionally (e.g., cold chain disruption) or intentionally (e.g., clabber) incubated at room temperature, retail milk samples were examined during a controlled RT incubation. During RT incubation, aerobic bacteria were significantly enriched in Raw, Vat, and HTST milk starting at 6 h, 12 h, and 24 h, respectively (LMM, *P* < 0.05, Fig. 1b). As a result, the abundance of aerobic bacteria was beyond the CDFA regulations at 12 h in raw and 24 h in Vat milk (Fig. 1b). We observed a significant enrichment of coliforms in raw milk beginning at 12 h (LMM, *P* < 0.001) and in Vat milk starting at 24 h (LMM, *P* < 0.05), which surpassed the CDFA regulations (Fig. 1c). No significant changes of coliform counts were observed in HTST and UHT milk (LMM, *P* > 0.05, Fig. 1c). Finally, the population of *E. coli* indicated a significant increase by the end of incubation (24 h) in raw milk (LMM, *P* < 0.001), while its abundance remained relatively stable in the other three types of milk (LMM, *P* > 0.05, Fig. 1d). In all cases, there was no measurable bacterial growth observed in UHT milk (LMM, *P* > 0.05, Fig. 1).

Given that raw milk is a potential source of food-borne pathogens, retail milk samples in the present study were screened for *Listeria* spp., *Salmonella enterica*, and *E. coli* O157:H7. None were detected. Indeed, a much larger sample size would be needed for a pathogen prevalence study, which was outside the scope of this work. However, of the isolated *E. coli* strains (*n* = 95), 84.2% (*n* = 80) possessed at least one antibiotic resistance phenotype, and 35.8% (*n* = 34) were multidrug resistant (≥ 2 resistance phenotypes). Ceftazidime resistance was the most prevalent phenotype in this cohort (*n* = 57; 60%) followed by resistance to amoxicillin (*n* = 24; 25.3%), tetracycline (*n* = 24; 25.3%), and streptomycin (*n* = 22; 23.2%) (Supplementary table 1). Genome sequencing of one representative multidrug-resistant *E. coli* strain JXLQYF114666 indicated the presence of nine transferrable ARGs (*aph*(*3”*)*-Ib*, *aph*(*6*)*-Id*, *bla*_CMY-2_, *bla*_TEM-1B_, *mdf*(*A*), *catA2*, *sul2*, *tet*(*B*), and *dfrA14*) conferring resistance to seven classes of medically important antibiotics (Supplementary table 2). The *bla*_CMY-2_ gene, known to confer high-level ceftazidime resistance [25], appeared to reside on an IncI1 ST12 plasmid in JXLQYF114666. Conjugation assays demonstrated ceftazidime-resistance was successfully transferred to azide-resistant *E. coli* strain J53 and ceftazidime-susceptible *Pseudomonas aeruginosa* strain UCDDAM001 at frequencies of 2.8 ± 0.35 (mean ± s.d.) × 10^–4^ and 6 ± 1.44 × 10^−7^ cells per recipient, respectively. In summary, bacterial populations in retail milk remained stable at refrigerated temperatures and did not contain known pathogens; however, RT incubation increased bacteria beyond state standards in raw and Vat-pasteurized milk and, even at cold temperatures, contained *E. coli* strains harboring a ceftazidime resistance gene which was transferrable.

### The dynamics of the retail milk microbiome in California during 4 °C and RT incubations

The bovine raw milk microbiota has been relatively well-studied on farms [17, 18], in tanker trucks, and in processing facilities [19], but the microbiome of retail raw and pasteurized milk, remain elusive. To address this gap, 16S rRNA gene sequencing was used to systematically characterize the microbiota of California retail milk. As expected due to differences in live bacterial populations, we obtained different numbers of reads across types of milk, in which raw (median 6062; interquartile range (IQR), 31,578) and Vat milk (median 1678; IQR, 40,718) had a similar high amount of sequences per sample followed by HTST (median 662; IQR, 2683) and UHT (median 264; IQR, 1395) milk. Sequencing quality did not vary systematically across types of retail milk in California (and raw milk across states) (Kruskal-Wallis, *P* = 0.3; Supplementary figure 2c & d).

**Fig. 2:**
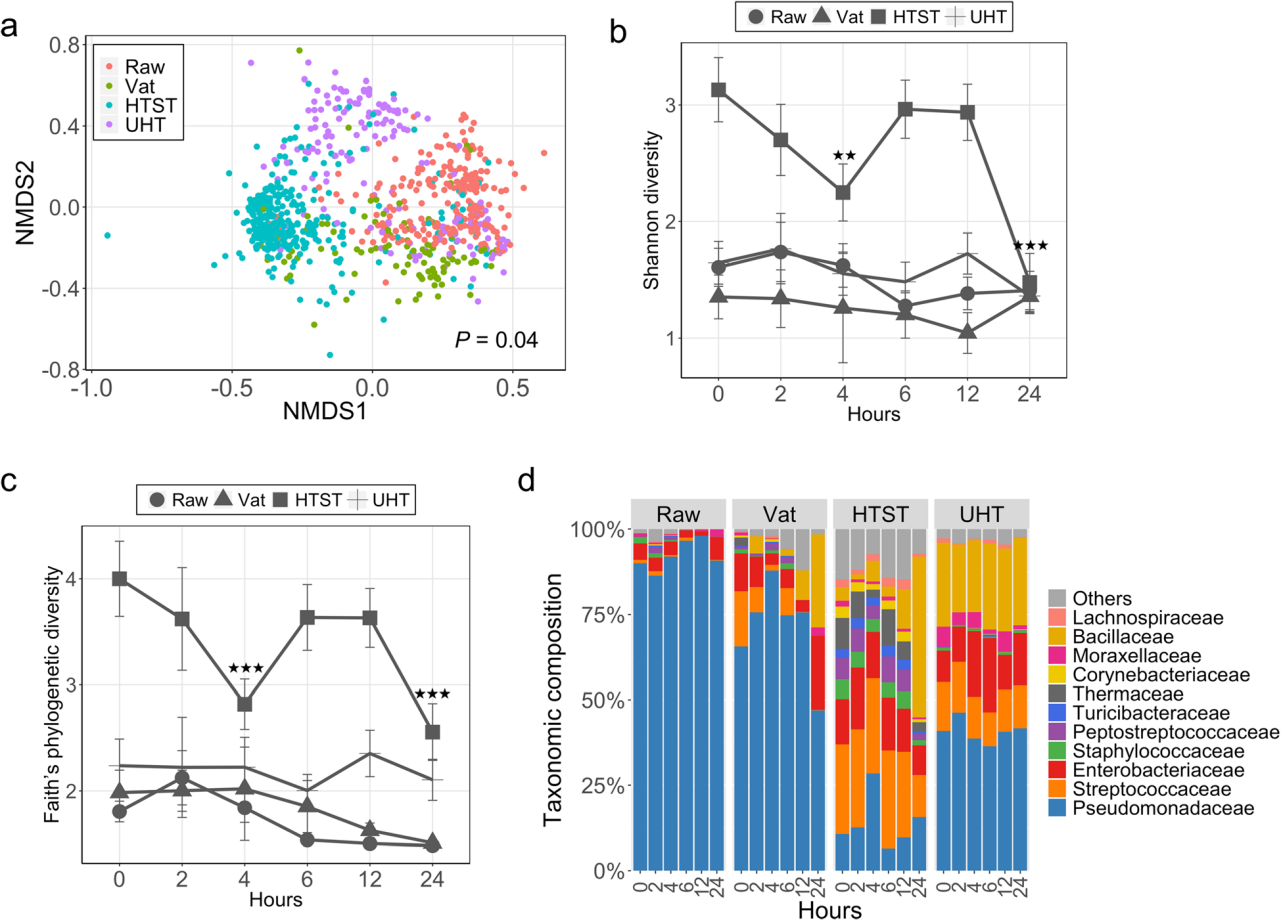
The microbiota profiles of retail milk over room temperature incubations. **a** NMDS of retail milk samples based on Bray-Curtis (*k* = 3; stress = 0.15; linear fit, *R*^2^ = 0.76; non-metric fit, *R*^2^ = 0.96). All 902 samples, after excluding low sequencing read milks (Raw = 270; Vat = 108; HTST = 347; UHT = 177), were included in this analysis. **b** Alpha diversity as measured by Shannon index in retail milk over RT incubation. **c** Alpha diversity as measured by Faith’s phylogenetic diversity (PD) in retail milk over RT incubation. **d** Bar plot depicting the relative abundance of bacterial families over time; bacterial families which has a relative abundance less than 1% were grouped into “Others”. ^★★^*P* < 0.01, and ^★★★^*P* < 0.001 for comparison with start point (0 h) in HTST milk

Overall, retail milk of different processing types possessed varied microbiota structure (Bray-Curtis, PERMANOVA test by adonis2; *P* = 0.04), in which HTST and UHT samples clustered in independent groups while raw and vat milk overlapped (Fig. 2a). Raw, Vat, and UHT milk samples indicated comparable alpha diversity, as measured by both Shannon index and Faith’s phylogenetic diversity (PD) which remained relatively stable throughout the RT incubation (LMM, *P* > 0.05, Fig. 2b and c). HTST milk microbiota had the highest diversity (LMM, *P* < 0.001 for both Shannon index and PD, Fig. 2b and c). This is consistent with the fact that HTST milk commonly comes from multiple dairies and bacteria that remain after heat-treatment are likely also diverse [26, 27]. Interestingly, the diversity estimated in HTST milk fluctuated over the RT incubation, during which both alpha diversities exhibited decreases at 4 h and 24 h (LMM, *P* < 0.01, Fig. 2b and c). In addition, we likely underestimated the alpha diversity of HTST milk as the subsampling depth of 200 reads was not able to capture all of its observed amplicon sequence variants (ASVs) (Supplementary figure 2b).

The microbiota variances were further investigated by examining the taxonomic composition. *Pseudomonadaceae* was dominant in Raw (> 90%; averaged relative abundance), Vat (> 70%), and UHT (> 45%) milks, while *Streptococcaceae* was more prevalent in HTST milk samples (Fig. 2d). The dominance of *Pseudomonadaceae* is consistent with lower temperature creating a selective advantage for psychrotolerant *Pseudomonas* spp. [28, 29]. Similar differences in microbiota were observed in retail milk kept in the cold environment, but the dynamic changes of alpha diversity were attenuated in HTST milk over the incubation compared to its dramatic change during RT incubation (Supplementary figure 3). Taken together, retail milk in California that underwent different processing and pasteurization procedures possessed distinct microbiomes. In addition, while viable bacterial populations measured in raw milk experienced a dramatic increase once incubated at RT, the relative abundance of the various taxa of raw milk, as observed by 16S amplicon sequencing, remained stable over the same incubation.

### The antibiotic resistome of California retail milk during RT incubations

To gain a deeper understanding of the milk resistome, shotgun metagenomic sequencing of DNA extracted from selected milk samples from California was conducted, garnering 109 Gb of sequencing data from 13 raw and 11 HTST milk microbiomes, with 6.5 (median; IQR, 2) million 150 bp paired-end (PE) reads per sample. Vat milk, of which the microbiota overlapped with raw milk (Fig. 2a), and UHT milk, in which no detectable live bacteria were observed (Fig. 1), were excluded from this analysis. Sequencing depth and quality did not vary significantly across types of milk on HiSeq 4000 (Supplementary figure. 4a & b).

**Fig. 3:**
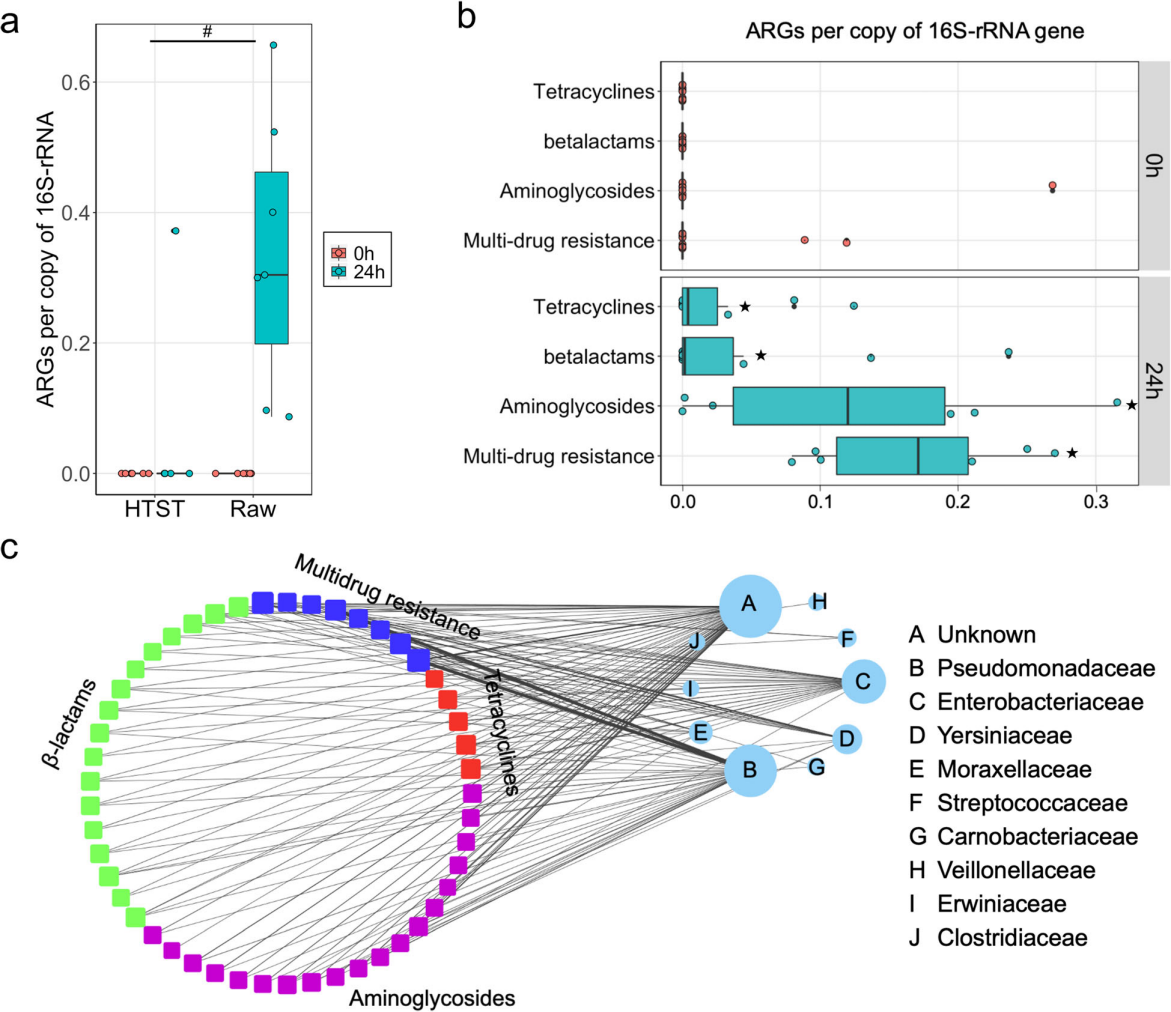
The resistome profile of California retail milk before and after RT incubation. **a** Within the sequencing data from HiSeq 4000, ARGs were undetectable prior to RT incubation in both HTST (*n* = 5) and raw (*n* = 6) milk samples. The prevalence of ARGs significantly increased in all RT-incubated raw milk (*n* = 7), with a single HTST sample (out of 6) possessing measurable ARGs. A Wilcoxon rank sum test was used for statistical comparison of the abundance of ARGs between HTST and raw milks (^#^*P* < 0.05). Boxplots denote the interquartile (IQR) between the first and third quartiles (25th and 75th percentiles, respectively) and median is denoted by the horizontal line. **b** RT incubation enriched the ARGs conferring resistance to 4 classes of antibiotics. A balanced design of raw milk samples before and after RT incubation from both HiSeq 4000 (*n* = 13) and NovaSeq S4 (*n* = 8) were included in this analysis. A Wilcoxon signed-rank test was used to assess the significant changes of ARG abundance belonging to each class of antibiotics, and an FDR correction was applied for multiple comparisons (^★^*P* < 0.05). **c** The observed ARGs in raw milk (*n* = 21) were predicted originate from 9 different bacterial families. Edge thickness indicates the normalized abundance of ARGs (rounded squares; colored by class of antibiotics) from a predicted bacterial family (circles). Node size represents the number of connections (degree). Detailed information of ARGs and bacteria networks is available in Supplementary Data 2.

ARGs were undetected in both HTST and raw milk samples at the first timepoint (0 h; Fig. 3a); however, the 24 h RT-incubation dramatically increased the measurable ARGs in raw milk microbiomes by comparison to HTST milk (Wilcoxon rank-sum test, *P* = 0.02, Fig. 3a). Specifically, ARGs were observed in all incubated raw milks but the presence of ARGs only occurred in a single HTST sample (Fig. 3a). Leveraging a much higher sequencing depth with NovaSeq S4, we were able to detect ARGs in raw milks without incubation, and in both sequencing runs (i.e., HiSeq and NovaSeq), ARGs were significantly enriched via incubation (Wilcoxon signed-rank test, *P* = 0.003, Fig. 3b). In total, 49 ARGs belonging to 15 ARG groups, representing 7 antibiotic resistance mechanisms were found in California retail raw milk samples (Supplementary Data 1). These ARGs were predicted to confer resistance to 4 classes of antibiotics in a normalized abundance (hereafter referred to as abundance) ranging from 0 to 0.66 copies of ARG per 16S rRNA gene in each sample (Fig. 3b). RT incubation significantly enriched ARGs belonging to all 4 classes of antibiotics (i.e., multidrug resistance, aminoglycosides, beta-lactams, and tetracyclines) within 24 h (Wilcoxon signed-rank test, *P* < 0.05, FDR correction for multiple comparison, Fig. 3b).

Metagenomic assembly was employed to track the bacterial host of the observed ARGs [30]. Overall, we obtained 18,940 (median; IQR, 14,579) contigs per sample, and the majority of ARG-containing reads (> 99%) were successfully aligned to assembled contigs (Supplementary figure 5a and b). Importantly, most observed ARGs (62-80%) were assigned a bacterial taxonomy at the family level (Supplementary figure 5c and d). Specifically, nine known bacterial families were predicted to host these ARGs, with *Pseudomonadaceae* harboring the highest number of unique ARGs (36) followed by *Enterobacteriaceae* (28), *Yersiniaceae* (14), and *Moraxellaceae* (8) (Fig. 3c; Supplementary Data 2). Therefore, retail raw milk in California was clearly a source of ARGs conferring resistance to 4 classes of medically important antibiotics; raw-milk-borne ARGs were readily amplified during RT-incubation and were mostly from *Pseudomonadaceae*, *Enterobacteriaceae*, and *Yersiniaceae*.

### The geographical variances of antibiotic resistome in retail raw milk across states

Given that milk microbes could originate from animal skin, feces, and the local environment [31], raw milk samples from different geographical locations likely possess a regional-specific resistome [32]. To assess such variances in the resistome of retail raw milk, a separate set of samples collected across the USA were subjected to shotgun metagenomic sequencing on the NovaSeq S4 platform. We obtained 589 Gb of sequencing data with 20 retail raw milk microbiomes from 4 states, approximately 40 (median; IQR, 5.9) million reads (150 bp PE) per sample. Consistent with previous observations, sequencing depth and quality remained comparable in raw milk samples across states (Supplementary figure 4c & d). For all shotgun metagenomic sequencing involved in this study (i.e., HiSeq and NovaSeq), we obtained sufficient sequencing depth to capture the bacterial species (profiled by Kraken2) and ARGs (identified by MEGARes) in retail milk samples (Supplementary figure 6).

**Fig. 4:**
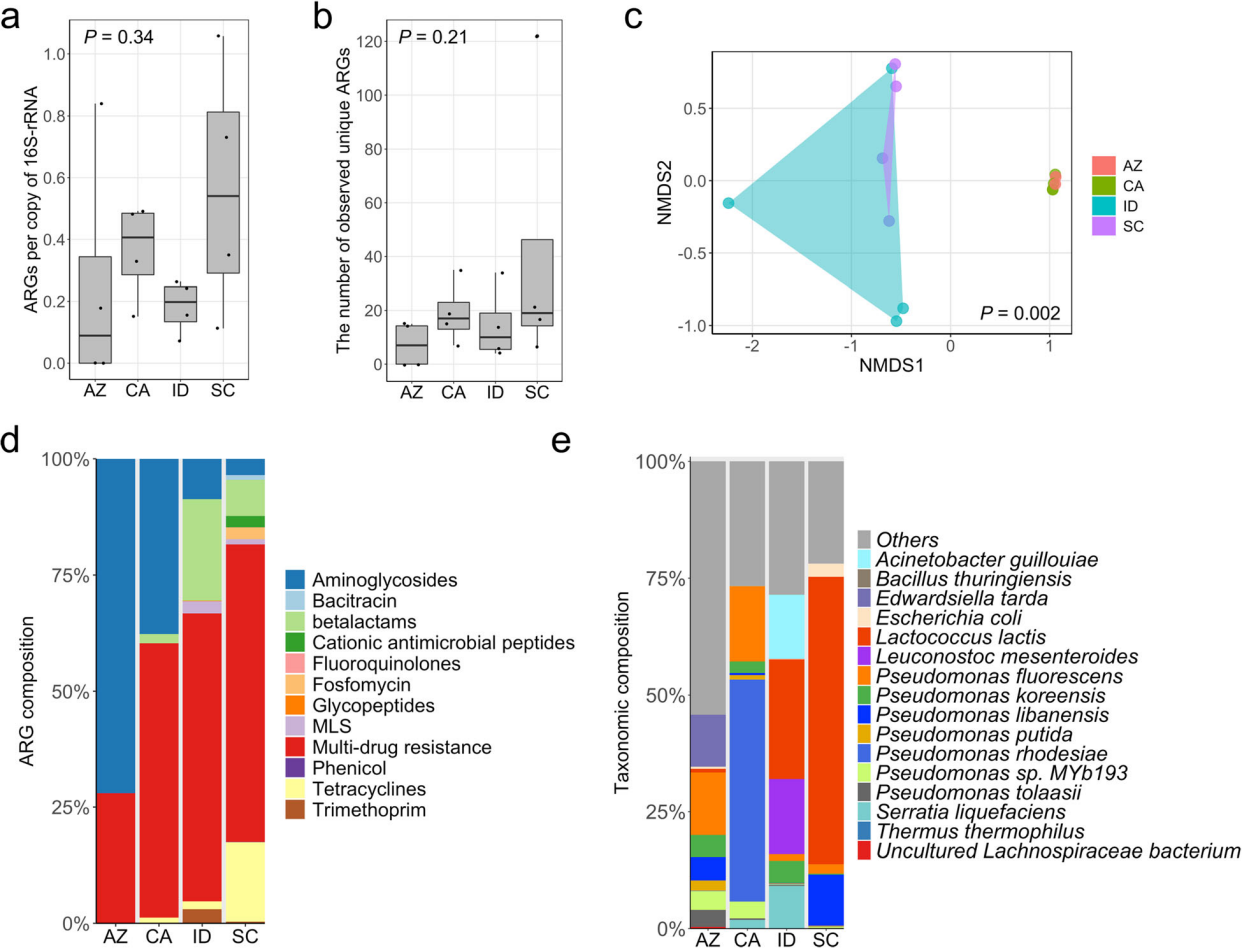
Geographical variance of raw milk resistomes in the USA. **a** The total normalized abundance of ARGs in raw milk samples (NovaSeq S4; *n* = 16) from different states. **b** The number of observed unique ARGs in raw milk samples (*n* = 16) from different states. **c** NMDS of raw milk resistome based on a Bray-Curtis dissimilarity calculation (*k* = 2; stress = 0.04; linear fit, *R*^2^ = 0.99; non-metric fit, *R*^2^ = 0.99). Polygons were applied to raw milk samples collected from the same area. **d** Relative abundance of ARGs by class of antibiotics per sample. **e** Relative abundance of bacterial genus per raw milk sample across states. A Kruskal-Wallis test was implemented to assess the statistical differences of normalized abundance and richness across milks

While raw milk samples from different geographic locations had varied abundance of ARGs (Kruskal-Wallis, *P* = 0.34, Fig. 4a), the diversity of ARGs remained comparable (Kruskal-Wallis, *P* = 0.21, Fig. 4b). Overall, we observed distinct resistome structure in raw milk from different states (Bray-Curtis, PERMANOVA test by adonis2; *P* = 0.002, Fig. 4c). Within the cohort of 4 distinct sampling areas, 176 unique ARGs were identified that confer resistance to 12 classes of antibiotics (Fig. 4d; Supplementary Data 1 and Data 3). ARGs were observed in 2 (out of 4) raw milk samples from Arizona, in which the ARG-containing raw milk samples resembled the resistome structure to those in California (Fig. 4c and d). In these samples, resistomes were dominated by ARGs in "multidrug resistances" and aminoglycosides classes (Fig. 4d). Raw milk samples from South Carolina and Idaho shared similarities in resistome content. In both states, “multidrug resistance” was the prominent class of ARGs, and ARGs conferring resistance to beta-lactams were more prevalent than observed in Arizona and California (Fig. 4d). Tetracyclines-related ARGs were more abundant in samples from South Carolina and ARGs to trimethoprim were more frequently observed in raw milk samples from Idaho (Fig. 4d). In general, the differences observed in resistomes of raw milk can be largely explained by variances observed in milk microbiota (Fig. 4e and Supplementary figure 7). Specifically, there were 123 different ARGs detected in raw milks from South Carolina belonging to 15 bacterial families, 43 ARGs observed in raw milks from Idaho belonging to 15 bacterial families, and 15 unique ARGs originating from 3 bacterial families observed in raw milk samples from Arizona (Supplementary figure 8, 9 and 10; Supplementary Data 2). In summary, retail raw milk samples taken through RT incubations were found to harbor ARGs conferring resistance to clinically relevant antibiotics; however, the type of ARGs differed by region.

## Discussion

Raw milk is commonly consumed worldwide [12, 22]. In the United States, raw milk is often promoted for various nutritional and protective benefits. Consumption of raw milk as a general practice has been criticized due to the concern over contamination by food-borne pathogens [7–11]. The increasing prevalence of antibiotic resistance (AMR) is a global concern [33, 34], and the role of raw milk in the dissemination of AMR is unclear. To address this knowledge gap, we implemented an integrated approach employing culture-omics and high-throughput sequencing to assess the dynamic changes of microbiome and resistome in raw milk over incubations as well as their difference from pasteurized retail milk. Our results indicate that raw milk is a clear source of ARGs, which are readily enriched during RT-incubations, and the antibiotic resistome varies significantly in raw milk across states.

The microbial and ARG content of clabber milk—raw milk incubated at room temperature for several days—likely varies by different milk sources, varied incubation time (1-5 days) and seasonally dependent room temperatures. However, the results presented here suggest that spontaneous fermentation does not grow beneficial lactic acid bacteria and instead, enrichment of ARGs occurred even within a short period of RT incubation. In addition to ARGs enriched due to intentional RT fermentation, proper cold-chain maintenance can fail during transportation from the raw milk producer to the consumer or in the home of the consumer, and therefore inadvertent short-term RT incubations can also happen. Such incubation likely enriches populations of bacteria in milk which in turn contributes to a modified resistome with elevated prevalence of ARGs. A better understanding the diversity of ARG content as well as the biogeography, health risk associations and methods to reduce this ARG reservoir, is clearly warranted.

Our findings also have implications for low-income countries, where consumption of unpasteurized dairy products is common [35, 36]. One recent study evaluated the risk factors associated with carriage of antibiotic-resistant *E. coli* from people in northern Tanzania, and direct microbial transmission in raw milk was found to be the primary predictor of the prevalence of AMR [12], highlighting the role of raw milk in maintaining reservoirs of ARGs and transmitting antibiotic resistance.

While comprehensive, our findings have limitations. In light of the low-biomass nature of milk samples, the inferred microbial ecology from high-throughput sequencing is often mixed with various contaminants [37]. We employed a relatively stringent data filtration (excluded samples with less than 200 reads) in our 16S rRNA gene sequencing dataset to reduce false positives. As expected, in the removed milk samples (*n* = 395), a large proportion (median 66.8%; IQR, 64.9%) of the microbiome were composed of shared ASVs with extraction controls, indicating potential DNA contaminants (Supplementary figure 2a). It is therefore possible that we underestimated true positives given low-sequencing reads may have been artificially excluded. Also, while metagenomic assembly offered us a unique opportunity to connect ARGs to a bacterial host [30], there were ARGs not assigned to any known bacterial families, which may be explained by the presence of chimeric contigs, and limitations of databases or taxonomy assignment software. Further, to rule out potential false-positive identification of ARGs which require single nucleotide changes to confer resistance, we implemented RGI [38]. Only ARGs which survived this confirmation were reported in this study. This conservative approach may also underestimate the diversity of ARGs (i.e., increases the rate of false negatives) present in retail milk if the short sequencing reads did not capture the SNP required to confer resistance [39].

In summary, our findings suggest that retail raw milk has a higher prevalence of ARGs than pasteurized milk, an effect amplified by RT incubation. Unlike the implied dominance of “beneficial” lactic acid bacteria in unpasteurized milk [40, 41], retail raw milk microbiota varied in samples from distinct geographical locations, possessed few lactic acid bacteria, and were frequently dominated with microbes within the family *Pseudomonadaceae*. Our findings suggest that retail raw milk is a clear source of antibiotic-resistant bacteria and ARGs. This potential public health hazard appears to be more prevalent than specific pathogens with documented presence in retail samples across brands and regions of the USA. The public health and medical communities should continue to inform consumers of the microbial food safety risks from consumption of raw milk, and the increased risk from temperature abuse.

## Conclusion

Employing a nation-wide retail milk sampling, we systematically studied the raw and pasteurized milk microbiota and antibiotic resistome. Raw milk microbiota and resistomes differ across geographical locations, and despite commonly advertised probiotic effects, raw milk possessed a limited component of lactic acid bacteria and is frequently dominated with *Pseudomonadaceae*. Compared to pasteurized milk, raw milk has a distinct microbiota with a higher abundance of viable bacteria containing antimicrobial resistance genes, both of which are amplified by spontaneous fermentation at room temperature. This work indicates that raw milk consumption poses an additional risk to consumers through transfer of antimicrobial resistance genes.

## Materials and methods

### Retail milk sampling, experimental design, and DNA extraction

The initial retail milk sampling occurred between March and August 2017 from grocery stores in California. A total of eight milk brands including ultra-pasteurized milk (UHT; 280-300 °F, 2-6 s; *n* = 2 brands), HTST pasteurized milk (high-temperature short-time, HTST; 161-165 °F,15-20 s; *n* = 3 brands), vat pasteurized milk (Vat; 145 °F, 30 min; *n* = 1 brand), and unpasteurized milk (raw milk, Raw, *n* = 2 brands) were examined in this study. All milk recruited via the initial sampling was whole milk which is certified as organic and rBST free (Supplementary table 3). Samples were collected from all brands of milk through eight independent purchases (biological replicates). For each purchase, the samples were aliquoted into three 15 mL tubes with 10 mL each (technical replicates). At 4 °C, milk samples were incubated for 0, 2, 4, 6, and 24 h; at 23 °C, milks experienced incubations for 0, 2, 4, 6, 12, and 24 h. Consequently, after incubation, a total of 1920 milk samples were obtained in California for analysis (Table 1).

**Table 1:** Sampling scheme and experimental design

Sampling states	Milk type	Incubation temperature	Incubation time	No. of milk brands	No. of samples	Bacterial plating	16S^a^	SMS^b^ (HiSeq 4000)	SMS^b^ (NovaSeq S4)
California	Raw	4 °C, 23 °C	0 h, 2 h, 4 h, 6 h,12h^c^, 24 h	2	480	✓	✓	✓ (*n* = 13)	✓ (*n* = 8)
	Vat	4 °C, 23 °C	0 h, 2 h, 4 h, 6 h,12 h, 24 h	1	240	✓	✓	✕	✕
	HTST	4 °C, 23 °C	0 h, 2 h, 4 h, 6 h,12 h, 24 h	3	720	✓	✓	✓ (*n* = 11)	✕
	UHT	4 °C, 23 °C	0 h, 2 h, 4 h, 6 h,12 h, 24 h	2	480	✓	✓	✕	✕
South Carolina	Raw	23 °C	0 h, 24 h	2	24	✕	✓	✕	✓ (*n* = 4)
Arizona	Raw	23 °C	0 h, 24 h	2	30	✕	✓	✕	✓ (*n* = 4)
Idaho	Raw	23 °C	0 h, 24 h	2	36	✕	✓	✕	✓ (*n* = 4)
Maine	Raw	23 °C	0 h	2	24	✕	✓	✕	✕

For samples from California, the incubations at different temperature shared a single start point (0 h). ✓ indicates all the collected samples were subjected to a type of analysis unless a number is specified, while ✕ represents the corresponding analysis was not performed. For the HiSeq 4000 run, 13 raw milk samples (6 samples were collected before any incubations and the remaining 7 samples experienced a 24 h incubation at 23 °C) and 11 HTST samples (5 samples were recruited before incubation and the other 6 were incubated at 23 °C for 24 h) were included to determine the resistome variances between milks as well as before and after room temperature incubation. Unequal number of metagenomes were obtained (i.e., 13, 11) as a result of unexpected sequencing failure of certain DNA samples. In the NovaSeq S4 run, 4 raw milk samples after a 24 h incubation at 23 °C from each state (California, South Carolina, Arizona, and Idaho) were used to assess the geographical variance of milk resistomes, and another 4 samples before incubation from the California cohort were included to validate results between sequencing platforms/runs

^a^16S rRNA gene sequencing

^b^Shotgun metagenomic sequencing

^c^Samples from 12-h incubation under 4 °C were skipped for bacterial plating

To assess the geographical variance, a separate sampling of raw milk occurred between October 2018 and February 2019 in 4 other states including Idaho, Arizona, South Carolina, and Maine. Sampling states were chosen based on the availability of raw milk in retail stores [42] to maximize the geographical distribution of milk. At each state, two brands of raw milk produced from two different dairy farms were purchased at least twice from retail stores (Table 1). All samples collected were whole, non-homogenized milk, which are certified as organic and rBST free. Purchased raw milk was immediately aliquoted into 15 mL tubes, and half of the subsequent milk samples were directly placed in the freezer (−20 °C), and the remaining were incubated at RT for 24 h and then were stored at −20 °C. Upon completion of sampling, a total of 114 samples were delivered on dry ice for analysis (Table 1).

For all the collected samples, approximately 2 mL vortexed milk was centrifuged at 10,000×*g* for 10 min (4 °C) to separate cells and fat from whey [43]. The supernatant and the fat layer were removed, and the pellet was kept frozen (−20 °C) [30, 43] until DNA extraction with a ZR Fecal DNA MiniPrep kit (ZYMO, Irvine, CA, USA).

### Quantification of total aerobic bacteria, coliform, and *E. coli* in retail milk

In the California cohort, at 23 °C, we collected a total of 1152 samples with 144 samples per milk brand, and we obtained 960 milk samples from the 4 °C incubation with 120 samples per milk brand. At each temperature for a given incubation time, 1 mL milk samples (serial dilutions were applied when appropriate) were used to culture onto 3 M Petrifilm plate (3 M, Maplewood, MN, USA) at 37 °C incubator for 24 h to quantify the total aerobic bacteria populations, coliform, and *E. coli*. All numbers were recorded and transformed into log_10_ CFU per mL of milk prior to downstream analysis. Zero values were adjusted with the analytical detection limit of our assays, 6.6 CFU/mL for total aerobic counts and 0.33 CFU/mL for both coliform and *E. coli*, following the formula “substitution = log (RAND() × (detection limit))” in Excel (Microsoft Corp., Redmond, WA). Mean values were calculated from three technical replicates, representing the biological milk sample, for statistical analyses. A Kruskal–Wallis test was used to assess the statistical difference of bacterial populations in different types of milk prior to incubations, and a Dunn test in FSA package (0.8.24) was used for multiple comparisons [false discovery rate (FDR) *p* value adjustments]. A linear mixed model (LMM) from the lme4 package (version 1.1.21) was implemented to test for associations between the incubation time and the bacterial population in each type of milk. The *glht* function in multcomp package (version 1.4.10) in combination with the *lsm* function in lsmeans package (version 2.30.0) was used for intra- and intergroup pairwise comparisons (*p* values were adjusted with a single-step method).

### *E. coli* isolation and antibiotic susceptibility testing

In the course of bacterial quantification, if present, 1-2 *E. coli* isolates were collected randomly per milk sample which resulted in a total of 95 presumptive *E. coli* isolates from California. The bacterial species identities were further confirmed by examining the entire 16S rRNA gene via Sanger sequencing [44]. All isolates were subjected to antibiotic susceptibility testing with a breakpoint assay [12]. Briefly, MacConkey agar was prepared with 16 clinically relevant antibiotics (Supplementary Table 1) of fixed concentration, which was guided by the Clinical and Laboratory Standards Institute minimum inhibitory concentrations for *Enterobacteriaceae *[45]. Genomic DNA of an *E. coli* strain with a multidrug resistance phenotype (Ampicillin-Ceftazidime-Chloramphenicol-Ciprofloxacin-Penicillin G-Piperacillin-Streptomycin-Tetracycline), which was labeled as *E. coli* strain JXLQYF114666, was extracted using a DNeasy Blood & Tissue Kit (Qiagen, Hilden, Germany) and was subsequently sequenced on an Illumina MiSeq platform (Reagent kit v2; 250 bp PE) at UC Davis DNA Technologies & Expression Analysis Core. Genome sequencing reads were assembled into contigs using SPAdes (3.10.1) with default parameters. Assembled contigs were run through ResFinder (3.1, [46]) and PlasmidFinder (2.0, [47]) to assess the presence of acquired ARGs and plasmids in *E. coli* strain JXLQYF114666, respectively. The plasmid type of *bla*_CMY-2_-harboring contig (53,836 bp) was characterized by using pMLST-2. 0[47].

### Conjugation experiments

A conjugation assay [48] was performed to determine whether the *bla*_CMY-2_ gene was present on a conjugative plasmid, with *E. coli* strain JXLQYF114666 as the donor and azide-resistant *E. coli* J53 (ATCC BAA2731) and ceftazidime-susceptible *P. aeruginosa* strain UCDDAM001 as recipients. Briefly, separate cultures of donor and recipient were prepared aerobically overnight (37 °C, 220 r.p.m.) in LB broth. The following day, individual overnight cultures were inoculated into fresh LB (1:100) until an OD_600nm_ of 0.6-0.8. Then, equal amount of donor and recipient cultures (2 mL) were centrifuged for 5 min at 10,000×*g*, and the pellets were resuspended in 50 μL LB broth and mixed. The resultant suspension was spread onto a LB agar plate and incubated stationary overnight at 37 °C. *E. coli* transconjugants were selected on Mueller–Hinton agar plates containing ceftazidime (16 μg/mL) and sodium azide (150 μg/mL), and *P. aeruginosa* transconjugants were selected on *Pseudomonas* isolation agar (Hardy Diagnostics, Santa Maria, CA) with ceftazidime (16 μg/mL). The presence of *bla*_CMY-2_ gene was confirmed by PCR [49] and sequencing in transconjugants. Transfer frequencies were calculated as the number of transconjugants obtained per input recipient cell.

### 16S rRNA gene sequencing and data analysis

Duplicate DNA samples (out of three replicates) from the incubation assay in California (*n* = 1,280), together with raw milk DNA from other states (*n* = 114) and extraction controls (i.e., blanks without any biological samples added; *n* = 37) were prepared for 16S rRNA gene sequencing as previously described [30]. Briefly, the forward F515 primer which includes an eight-nucleotide barcode unique to each sample and a two-nucleotide linker sequence (5′-NNNNNNNNGTGTGCCAGCMGCCGCGGTAA-3′) and the reverse R806 primer (5′-GGACTACHVGGGTWTCTAAT-3′) were used to amplify the V4 region of the 16S rRNA gene. PCR reactions were carried out in triplicate in a 15-μL reaction containing 1X GoTaq Green Mastermix (Promega, Madison, WI, USA), 1 mM MgCl2 and 2 pmol of each primer. The PCR amplification conditions included an initial denaturation step of 2 min at 94 °C, followed by 25 cycles of 94 °C for 45 s, 50 °C for 60 s, and 72 °C for 90 s, followed by a final extension step at 72 °C for 10 min. Triplicate reactions were combined and purified using a Qiagen PCR purification column and submitted to the DNA Technologies & Expression Analysis Core at UC Davis for sequencing on an Illumina MiSeq platform (Reagent kit v2; 250 bp PE).

Raw sequencing reads were demultiplexed by using the Sabre software (*sabre pe*) (https://github.com/najoshi/sabre), and the sequencing quality was assessed by using FASTQC (version 0.11.9) per sequence [50]. In light of the number of sequences obtained from blank control samples (< 138 reads/sample), samples with low sequencing read counts (< 200 reads) were excluded, resulting in a total of 902 samples (Raw = 270; Vat = 108; HTST = 347; UHT = 177) for downstream analysis. In the remaining samples (*n* = 902), potential sequencing contaminants were further identified and removed using decontam (version 1.4.0; method = “prevalence”) with the default probability threshold [51]. Applying the same filtration criteria, in other states, 97 raw milk samples (AZ = 24; ID = 32; ME = 21; SC = 20) with 7458 (median; IQR, 15,796) sequences survived for further analysis. Our data filtration excluded a total of 395 retail milk samples (HTST = 146; UHT = 137; Raw = 69; Vat = 39), in which 66.8% (median; IQR, 64.9%) of sequencing reads represented shared ASVs with extraction controls (Supplementary figure 2a).

The remaining sequencing reads were then loaded into QIIME2 (version: 2019.1, [52]) (*qiime tools import --type ‘SampleData [PairedEndSequencesWithQuality]’ --source-format PairedEndFastqManifestPhred33*). The sequence quality control and feature table construction were performed using DADA2 [53] (*qiime dada2 denoise-paired --p-trim-left-f 21 --p-trim-left-r 23 --p-trunc-len-f 242 --p-trunc-len-r 250*). The feature table was rarefied at the maximum sampling depth of 200 reads, and was used to calculate the alpha diversity as measured by Shannon index and Faith’s phylogenetic diversity (*qiime diversity core-metrics-phylogenetic*). Our rarefaction curve analysis (*qiime diversity alpha-rarefaction*) indicated that a subsampling of 200 reads per sample was able to capture the majority of the observed ASVs in this cohort (Supplementary figure 2b). A linear mixed model (LMM) from the lme4 package [54] (version 1.1.21) was implemented to test for associations between the incubation time and the alpha diversity in each type of milk. The *glht* function in multcomp package [55] (version 1.4.10) in combination with the *lsm* function in lsmeans package [56] (version 2.30.0) was used for intra- and intergroup pairwise comparisons (*p* values were adjusted with a single-step method). Beta diversity was examined with Bray-Curtis distance matrices based on a CSS [57] normalized feature table and was visualized using non-metric multidimensional scaling (NMDS) in R [58]. Differences in beta-diversity were tested using adonis2 (PERMANOVA test) in the vegan package [59] after checking for differences in dispersion using betadisper. Taxonomy was assigned using QIIME2 (*qiime feature-classifier classify-sklearn*) against the SILVA database (release 132, [60]).

### Shotgun metagenomic sequencing

The initial metagenomic sequencing run with retail milk samples solely from California (raw = 13, HTST = 11) was completed on an Illumina HiSeq 4000 platform and the second sequencing run with raw milk samples from 4 states (*n* = 20) was performed on an Illumina NovaSeq S4 platform (Table 1). Both sequencing libraries were prepared following the same procedure in the UC Berkeley Functional Genomics Laboratory (FGL) and were sequenced with the 150 paired-end reads strategy in the Vincent J. Coates Genomics Sequencing Laboratory at the University of California, Berkeley.

Briefly, each sample was sheared using the 150 bp setting of the Diagenode Bioruptor, then purified and concentrated with the Qiagen Minelute cleanup kit. End repair, a tailing of DNA fragments, and adapter ligation were performed using the KAPA Hyper Prep library kit. Next, 9 cycles of indexing PCR were performed using the KAPA Hi-Fi Hotstart amplification kit. Cleanup and dual-SPRI size selection were completed using AMPure beads. Libraries were checked for quality on the AATI fragment analyzer.

Raw sequences were used to assess the sequencing quality by calculating the average quality score per sequence using FASTQC (version 0.11.9) [50]. To avoid host DNA contamination, BMTagger in bmtools (version 1) was used to remove reads aligning to the bovine genome (version UMD3.1) from all samples. The resulting reads were then trimmed using Trimmomatic (version 0.36) [61] and merged using FLASH (version 1.2.11) [62] prior to downstream analysis. Taxonomy profiling of metagenomes were performed using Kraken2 [63] to map against a custom database including RefSeq [64] and 4941 metagenome-assembled rumen genomes [65]. The relative abundance of the bacterial genus was estimated using Bracken [66].

### Antibiotic resistome analyses

Merged sequencing reads were aligned to the ARG database MEGARes (version 1.0.1) [67] using BWA with default settings [68]. In the generated SAM formatted file, alignments tagged with “RequiresSNPconfirmation” were extracted which were further subjected to a secondary functional validation by using Resistance Gene Identifier (RGI) (version 5.1.0) with “Perfect” and “Strict” algorithms [38]. This secondary analysis is critical to rule out false-positive ARGs which often require single nucleotide mutations to confer resistance [69]. Sequencing reads did not survive the RGI-based confirmation were excluded from the SAM file, and the remaining alignments were then analyzed through ResistomeAnalyzer (-t 80; at least 80% of nucleotides in the reference sequence that were aligned to by at least one sequence read) to quantify ARGs (https://github.com/cdeanj/resistomeanalyzer). This analysis outputs data into gene, group, mechanism, and class levels corresponding to the levels of the annotation in the database hierarchy [67], and the gene-level data (e.g., TEM-77, TEM-107, TEM-73, etc.) were used to calculate the ARG diversity. MEGARes (version 1.0.1) is a manually curated database that consists of a collection of 3824 ARGs with the reference sequences ranging in size from 211 to 4185 bp [67]. The counts data were normalized with 16S rRNA gene by including the information of ARG sequence length and sequencing depth. Normalized ARG abundance was expressed as “copy of ARG per copy of 16S rRNA gene” as suggested by Li et al. [30, 70, 71]. The number of reads mapping to 16S rRNA bacterial gene was determined using METAXA2 (version 2.1.3) [72], and 1432 bp was used for calculations as the average length of 16S rRNA gene. Differences in resistome structure across states based on Bray-Curtis distance measures were tested using adonis2 (PERMANOVA test) in the vegan package after checking for differences in dispersion using betadisper.

Metagenome assemblies were generated with trimmed but un-merged reads for each sample using MEGAHIT (version 1.0.6) with default parameters [73]. The assembled contigs were used to predict the bacterial origin of observed ARGs. Specifically, the ARG-aligned sequencing reads, which survived the RGI confirmation, were used to align to contigs with BWA-MEM [68], and contigs that contain ARG sequences were kept for taxonomic assignment. taxator-tk (version 1.3.3) [74], a software designed to perform taxonomic analysis of assembled metagenomes, was applied to predict the bacterial origin of contigs. In particular, we used taxator-tk with our custom database which includes both RefSeq [64] and 4941 rumen-related metagenome-assembled genomes [65] with parameters -a megan-lca -t 0.3 -e 0.01 to assign taxonomy of ARG-containing contigs at the family level. All networks were visualized using Cytoscape (version 3.7.2) [75].

## Supplementary information

**Additional file 1: Supplementary Data 1.** List of ARGs observed in milk samples from California.

**Additional file 2: Supplementary Data 2.** Data employed in all network analyses in this work.

**Additional file 3: Supplementary Data 3.** List of ARGs observed in raw milk from Arizona, Idaho, and South Carolina.

**Additional file 4: Supplementary Table 1.** Antibiotics and minimum inhibitory concentration used for the breakpoint assay. All 95 *E. coli* strains isolated from retail raw milk in California were subjected to the antibiotic susceptibility testing with a collection of 16 clinically relevant antibiotics.

**Additional file 5: Supplementary Table 2.** Antibiotic resistance genes detected from whole-genome sequencing of *E. coli* strain JXLQYF114666.

**Additional file 6: Supplementary Table 3.** Metadata of retail milk samples collected in California. This retail milk sampling occurred between March and August 2017 from grocery stores in California.

**Additional file 7: Supplementary Figure 1**. The bacterial population in retail milk over incubations at 4°C. **a**, Total aerobic bacteria **b**, Coliform **c**, *E. coli* populations during the 4°C incubation within 24h. A total of 960 milk samples from California were analyzed; Raw (n=240), HTST (n=360), Vat (n=120), and UHT (n=240). Solid and dashed horizontal lines (red) represent the California milk limit for aerobic bacteria (< 15,000 bacteria/mL) and coliform (< 10 coliform/mL), respectively. A linear mixed model (LMM) from the lme4 package (version 1.1.21) in R was implemented to test for associations between the incubation time and the bacterial population in each type of milk. ★*P* < 0.05 for comparison with start point (0 hour) in raw milk.

**Additional file 8: Supplementary Figure 2**.The descriptive analysis of 16S rRNA gene sequencing in retail milk. **a**, The relative abundance of 15 shared ASVs (with extraction controls) in excluded low-read milk samples (n=395). Milk samples were ranked by sequencing depth. **b**, The rarefaction curve of observed ASVs across types of retail milk in California. The vertical orange line indicates the subsampling read depth for rarefied feature table. **c**, The distribution of sequencing reads by phred quality score in retail milk from California. **d**, The distribution of sequencing reads by phred quality score in raw milk across states in the United States. A Kruskal-Wallis test was used to assess the statistical significance of sequencing quality variance.

**Additional file 9: Supplementary Figure 3**. The microbiota profiles of retail milk over 4°C incubations. **a**, alpha diversity as measured by Shannon index in retail milk over 4°C incubation. **b**, alpha diversity as measured by Faith's phylogenetic diversity (PD) in retail milk over 4°C incubation. **c**, Bar plot depicting the relative abundance of bacterial families over time; bacterial families which has a relative abundance less than 1% were grouped into “Others”. A linear mixed model (LMM) from the lme4 package (version 1.1.21) was implemented to test for associations between the incubation time and the alpha diversity in each type of milk. ★*P* < 0.05 for comparison with start point (0 hour) in HTST milk.

**Additional file 10: Supplementary Figure 4**. The descriptive analysis of shotgun metagenomic sequencing in retail milk. **a**, The sequencing depth of HTST and raw milk samples in California sequenced via HiSeq. **b**, The distribution of sequencing reads by phred quality score in retail milk from California. **c**, The sequencing depth of raw milk samples sequenced via NovaSeq across states. **d**, The distribution of sequencing reads by phred quality score in raw milk across states in the United States. A Kruskal-Wallis test was used to assess the statistical significance of sequencing depth and quality variances.

**Additional file 11: Supplementary Figure 5**. Descriptive analyses of metagenomic assembly and taxonomic assignment of ARGs. All raw milk samples harboring ARGs (n=23) were included in this analysis. **a**, Boxplot depicting the distribution of number of metagenomic assembled contigs in raw milk across states. A Kruskal-Wallis test was used to assess the statistical significance of contigs between states. **b**, The percentage of ARG-containing sequencing reads which were successfully aligned to metagenomic assembled contigs. **c**, The percentage of ARG-containing contigs assigned at the family level. **d,** The percentage of ARG-containing reads assigned at the family level.

**Additional file 12: Supplementary Figure 6**. Rarefaction curves of taxonomic profiling (kraken 2) and resistome analysis (MEGARes) in retail milk. **a**, The rarefaction curve of kraken 2 observed bacterial species in raw milks samples (n=16). Only raw milk samples sequenced via NovaSeq and used for taxonomic comparisons in Figure 4e were included in this analysis. **b**, The rarefaction curve of observed ARGs via MEGARes in retail milk across states. All retail milk samples harboring ARGs in this cohort (n=24; Raw=23, HTST=1) were included in this analysis.

**Additional file 13: Supplementary Figure 7**. The microbiota of retail raw milk from five states in the United States. **a**, alpha diversity as measured by Shannon index in raw milk across states. **b**, alpha diversity as measured by Faith's phylogenetic diversity (PD) in raw milk across states. **c**, NMDS of raw milk samples based on Bray-Curtis (k=3; stress = 0.17; Linear fit, R2=0.80; Non-metric fit, R2=0.97). Subsampling to obtain equal sample size (n = 20) for each state was completed prior to performing the ordination analysis and PERMANOVA. The centroid of each ellipse represents the group mean, and the shape was defined by the covariance within each group. **d**, Bar plot depicting the relative abundance of bacterial families across states; bacterial families which has a relative abundance less than 1% were grouped into “Others”. **e,** Best taxonomic discriminators of retail raw milk microbiota across states ranked by random forest classifier (mean importance > 0.01). ASVs were ranked by a random forest classifier available in the randomForest package (4.6.14) in R. **a-b**, A Kruskal–Wallis test was used to assess the statistical difference of alpha diversity in raw milk from different states, and a Dunn test in FSA package (0.8.24) was used for multiple comparisons [false discovery rate (FDR) *p* value adjustments]. Different letters indicate statistically significant groups.

**Additional file 14: Supplementary Figure 8**. The predicted bacterial families of ARGs observed in raw milk from South Carolina. There were 123 ARGs originating from 15 bacterial families were detected in milk samples. Families harboring <10 individual ARGs were not labeled, and detailed information of ARGs and bacteria networks is available in Supplementary Data 2. Edge thickness indicates the normalized abundance of ARGs (rounded squares; colored by class of antibiotics) from a predicted bacterial family (circles). Node size represents the number of connections (degree).

**Additional file 15: Supplementary Figure 9**. The predicted bacterial families of ARGs observed in raw milk from Idaho. There were 43 ARGs originating from 15 bacterial families were detected in milk samples. Families harboring <10 individual ARGs were not labeled, and detailed information of ARGs and bacteria networks is available in Supplementary Data 2. Edge thickness indicates the normalized abundance of ARGs (rounded squares; colored by class of antibiotics) from a predicted bacterial family (circles). Node size represents the number of connections (degree).

**Additional file 16: Supplementary Figure 10**. The predicted bacterial families of ARGs observed in raw milk from Arizona. There were 15 ARGs originating from 3 bacterial families were detected in milk samples. Detailed information of ARGs and bacteria networks is available in Supplementary Data 2. Edge thickness indicates the normalized abundance of ARGs (rounded squares; colored by class of antibiotics) from a predicted bacterial family (circles). Node size represents the number of connections (degree).

## Data Availability

Sequencing data generated from both amplicon and shotgun metagenomes in this study have been deposited with the NCBI SRA (PRJNA575106) and are publicly available. The genome sequences of *E. coli* strain JXLQYF114666 was submitted to JGI and is available under Ga0374221. Other data of this study are available from the authors upon reasonable request.
