# Supplementary material for: Reservoirs of antimicrobial resistance genes in retail raw milk
Source: Microbiome. 2020 Jun 26;8:99. doi: 10.1186/s40168-020-00861-6 (PMC7320593; doi:10.1186/s40168-020-00861-6)
Supplement: Supplementary file 5 — Additional file 4: Supplementary Table 1. Antibiotics and minimum inhibitory concentration used for the breakpoint assay. All 95 E. coli strains isolated from retail raw milk in California were subjected to the antibiotic susceptibility testing with a collection of 16 clinically relevant antibiotics. [file 40168_2020_861_MOESM4_ESM.doc]

**Supplementary Table 1:** Antibiotics and minimum inhibitory concentration used for the breakpoint assay. All 95 *E. coli* strains isolated from retail raw milk in California were subjected to the antibiotic susceptibility testing with a collection of 16 clinically relevant antibiotics.

| Antibiotic | MIC breakpoints (µg/mL) | No. of positive isolates | Percentage |
| --- | --- | --- | --- |
| Ceftazidime | 16 | 57 | 60.0% |
| Amoxicillin | 32 | 24 | 25.3% |
| Tetracycline | 16 | 24 | 25.3% |
| Streptomycin | 16 | 22 | 23.2% |
| Ampicillin | 32 | 13 | 13.7% |
| Piperacillin | 16 | 9 | 9.5% |
| Penicillin G | 128 | 8 | 8.4% |
| Ciprofloxacin | 4 | 6 | 6.3% |
| Chloramphenicol | 32 | 6 | 6.3% |
| Rifampicin | 8 | 5 | 5.3% |
| Gentamicin | 16 | 4 | 4.2% |
| Colistin | 8 | 2 | 2.1% |
| Cefoxitin | 32 | 1 | 1.0% |
| Cefepime | 16 | 1 | 1.0% |
| Kanamycin | 64 | 1 | 1.0% |
| Meropenem | 4 | 0 | 0.0% |
