# Supplementary material for: Reservoirs of antimicrobial resistance genes in retail raw milk
Source: Microbiome. 2020 Jun 26;8:99. doi: 10.1186/s40168-020-00861-6 (PMC7320593; doi:10.1186/s40168-020-00861-6)
Supplement: Supplementary file 6 — Additional file 5: Supplementary Table 2. Antibiotic resistance genes detected from whole-genome sequencing of E. coli strain JXLQYF114666. [file 40168_2020_861_MOESM5_ESM.doc]

**Supplementary Table 2:** Antibiotic resistance genes detected from whole-genome sequencing of *E. coli* strain JXLQYF114666.

| Resistance gene | Resistance phenotype | Position^a^ | Site | % Identity^b^ | Accession no.^b^ |
| --- | --- | --- | --- | --- | --- |
| *aph(3'')-Ib* | Aminoglycosides | Ga0374221_036 | 6059-6862 | 99.88 | AF321551 |
| *aph(6)-Id* | Aminoglycosides | Ga0374221_036 | 6868-7698 | 100.00 | CP000971 |
| *bla*_CMY-2_ | β-Lactams | Ga0374221_022 | 27560-28705 | 100.00 | X91840 |
| *bla*_TEM-1B_ | β-Lactams | Ga0374221_048 | 36-896 | 100.00 | AY458016 |
| *mdf(A)* | MLS^c^ | Ga0374221_011 | 27100-28332 | 98.46 | Y08743 |
| *catA2* | Phenicol | Ga0374221_053 | 219-860 | 96.11 | X53796 |
| *sul2* | Sulfonamides | Ga0374221_036 | 5183-5998 | 100.00 | AY034138 |
| *tet(B)* | Tetracyclines | Ga0374221_037 | 1334-2539 | 100.00 | AF326777 |
| *dfrA14* | Trimethoprim | Ga0374221_055 | 134-607 | 100.00 | KF921535 |

Note: ^a^Whole-genome sequencing resulted in 121 contigs under GOLD Analysis Project Id: Ga0374221. ^b^Percent nucleotide identity and corresponding GenBank accession no. for reference sequence. ^c^MLS - Macrolide, Lincosamide and Streptogramin B.
