# Supplementary material for: Reservoirs of antimicrobial resistance genes in retail raw milk
Source: Microbiome. 2020 Jun 26;8:99. doi: 10.1186/s40168-020-00861-6 (PMC7320593; doi:10.1186/s40168-020-00861-6)
Supplement: Supplementary file 7 — Additional file 6: Supplementary Table 3. Metadata of retail milk samples collected in California. This retail milk sampling occurred between March and August 2017 from grocery stores in California. [file 40168_2020_861_MOESM6_ESM.docx]

**Supplementary Table 3:** Metadata of retail milk samples collected in California. This retail milk sampling occurred between March and August 2017 from grocery stores in California.

| Milk ID | Pasteurization | Homogenization | Whole Milk | rBST | Organic |
| --- | --- | --- | --- | --- | --- |
| A | Raw | No | Yes | Free | Yes |
| B | Raw | No | Yes | Free | Yes |
| C | Vat | No | Yes | Free | Yes |
| D | HTST | No | Yes | Free | Yes |
| E | HTST | Yes | Yes | Free | Yes |
| F | HTST | Yes | Yes | Free | Yes |
| G | UHT | Yes | Yes | Free | Yes |
| H | UHT | Yes | Yes | Free | Yes |
