# Supplementary material for: Reservoirs of antimicrobial resistance genes in retail raw milk
Source: Microbiome. 2020 Jun 26;8:99. doi: 10.1186/s40168-020-00861-6 (PMC7320593; doi:10.1186/s40168-020-00861-6)
Supplement: Supplementary file 7 [file 40168_2020_861_MOESM10_ESM.docx]

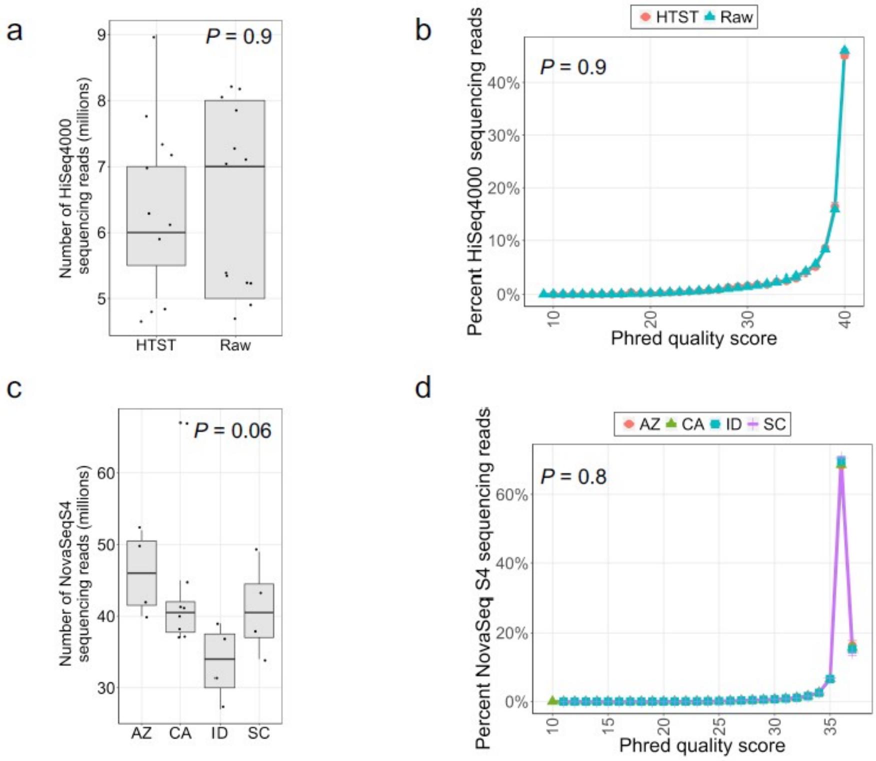

Supplementary Figure 4: The descriptive analysis of shotgun metagenomic sequencing in retail milk. **a**, The sequencing depth of HTST and raw milk samples in California sequenced via HiSeq. **b**, The distribution of sequencing reads by phred quality score in California retail milk. **c**, The sequencing depth of raw milk samples sequenced via NovaSeq across states. **d**, The distribution of sequencing reads by phred quality score in raw milk across states in the United States. A Kruskal-Wallis test was used to assess the statistical significance of sequencing depth and quality variances.
