# Supplementary material for: Reservoirs of antimicrobial resistance genes in retail raw milk
Source: Microbiome. 2020 Jun 26;8:99. doi: 10.1186/s40168-020-00861-6 (PMC7320593; doi:10.1186/s40168-020-00861-6)
Supplement: Supplementary file 8 [file 40168_2020_861_MOESM11_ESM.docx]

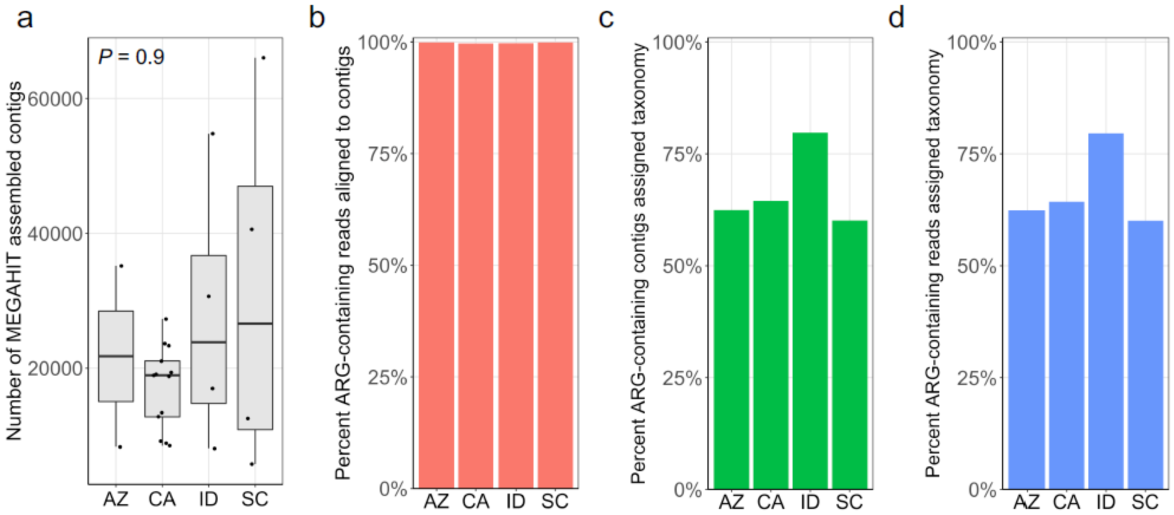

Supplementary Figure 5: Descriptive analyses of metagenomic assembly and taxonomic assignment of ARGs. All raw milk samples harboring ARGs (n=23) were included in this analysis. **a**, Boxplot depicting the distribution of number of metagenomic assembled contigs in raw milk across states. A Kruskal-Wallis test was used to assess the statistical significance of contigs between states. **b**, The percentage of ARG-containing sequencing reads which were successfully aligned to metagenomic assembled contigs. **c**, The percentage of ARG-containing contigs which were successfully assigned with a bacterial taxonomy at the family level. **d,** The percentage of ARG-containing reads which were successfully assigned with a bacterial taxonomy at the family level.
