# Supplementary material for: Reservoirs of antimicrobial resistance genes in retail raw milk
Source: Microbiome. 2020 Jun 26;8:99. doi: 10.1186/s40168-020-00861-6 (PMC7320593; doi:10.1186/s40168-020-00861-6)
Supplement: Supplementary file 9 [file 40168_2020_861_MOESM12_ESM.docx]

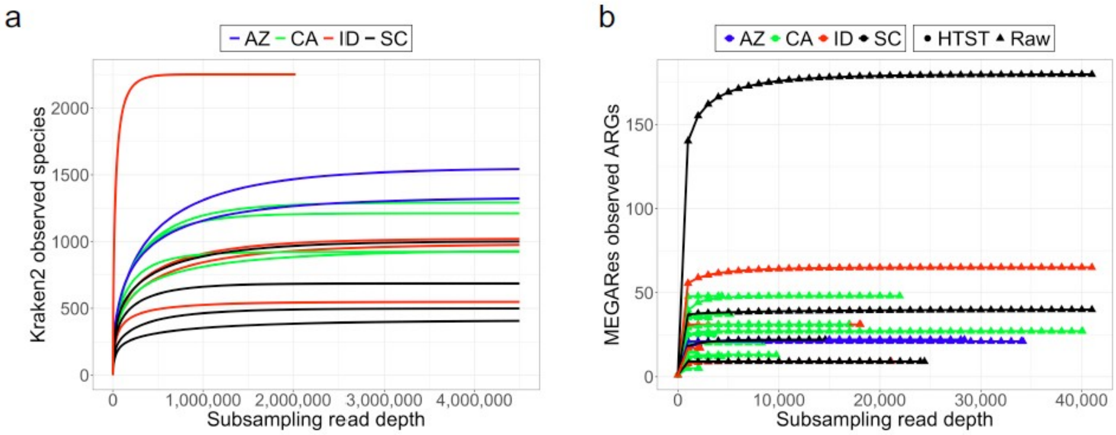

Supplementary Figure 6: Rarefaction curves of taxonomic profiling (kraken 2) and resistome analysis (MEGARes) in retail milk. **a**, The rarefaction curve of kraken 2 observed bacterial species in raw milks samples (n=16). Only raw milk samples sequenced via NovaSeq and used for taxonomic comparisons in Figure 4e were included in this analysis. **b**, The rarefaction curve of observed ARGs via MEGARes in retail milk across states. All retail milk samples harboring ARGs in this cohort (n=24; Raw=23, HTST=1) were included in this analysis.
