# Supplementary material for: Reservoirs of antimicrobial resistance genes in retail raw milk
Source: Microbiome. 2020 Jun 26;8:99. doi: 10.1186/s40168-020-00861-6 (PMC7320593; doi:10.1186/s40168-020-00861-6)
Supplement: Supplementary file 10 [file 40168_2020_861_MOESM13_ESM.docx]

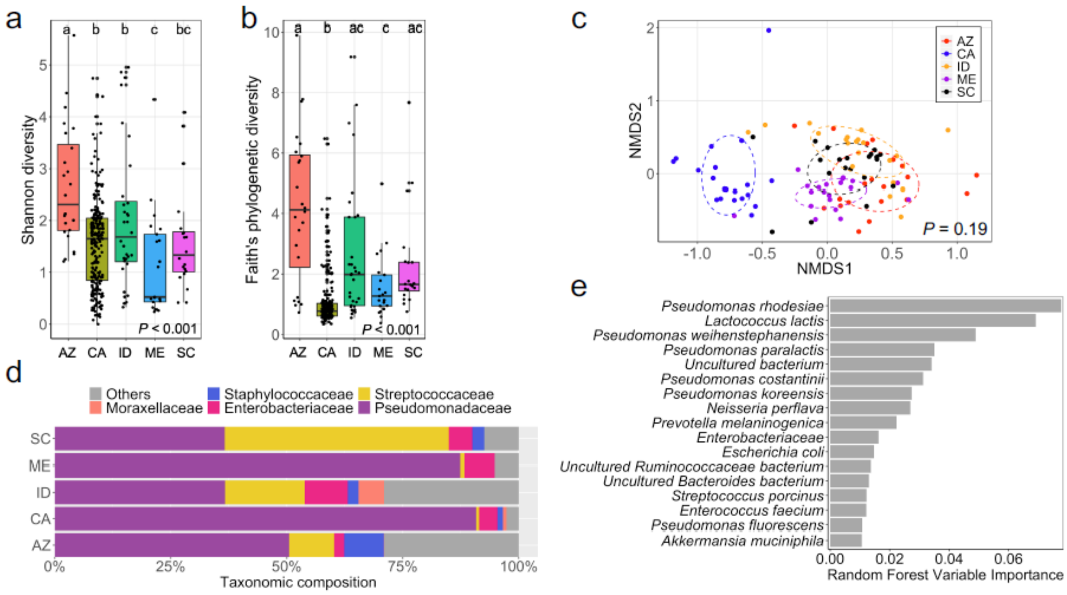

Supplementary Figure 7: The microbiota of retail raw milk from five states in the United States. **a**, alpha diversity as measured by Shannon index in raw milk across states. **b**, alpha diversity as measured by Faith's phylogenetic diversity (PD) in raw milk across states. **c**, NMDS of raw milk samples based on Bray-Curtis (k=3; stress = 0.17; Linear fit, R^2=0.80; Non-metric fit, R^2=0.97). Subsampling to obtain equal sample size (n = 20) for each state was completed prior to performing the ordination analysis and PERMANOVA. The centroid of each ellipse represents the group mean, and the shape was defined by the covariance within each group. **d**, Bar plot depicting the relative abundance of bacterial families across states; bacterial families which has a relative abundance less than 1% were grouped into "Others". **e,** Best taxonomic discriminators of retail raw milk microbiota across states ranked by random forest classifier (mean importance > 0.01). ASVs were ranked by a random forest classifier available in the randomForest package (4.6.14) in R. **a-b**, A Kruskal–Wallis test was used to assess the statistical difference of alpha diversity in raw milk from different states, and a Dunn test in FSA package (0.8.24) was used for multiple comparisons [false discovery rate (FDR) *p* value adjustments]. Different letters indicate statistically significant groups.
