# Supplementary material for: Reservoirs of antimicrobial resistance genes in retail raw milk
Source: Microbiome. 2020 Jun 26;8:99. doi: 10.1186/s40168-020-00861-6 (PMC7320593; doi:10.1186/s40168-020-00861-6)
Supplement: Supplementary file 11 [file 40168_2020_861_MOESM14_ESM.docx]

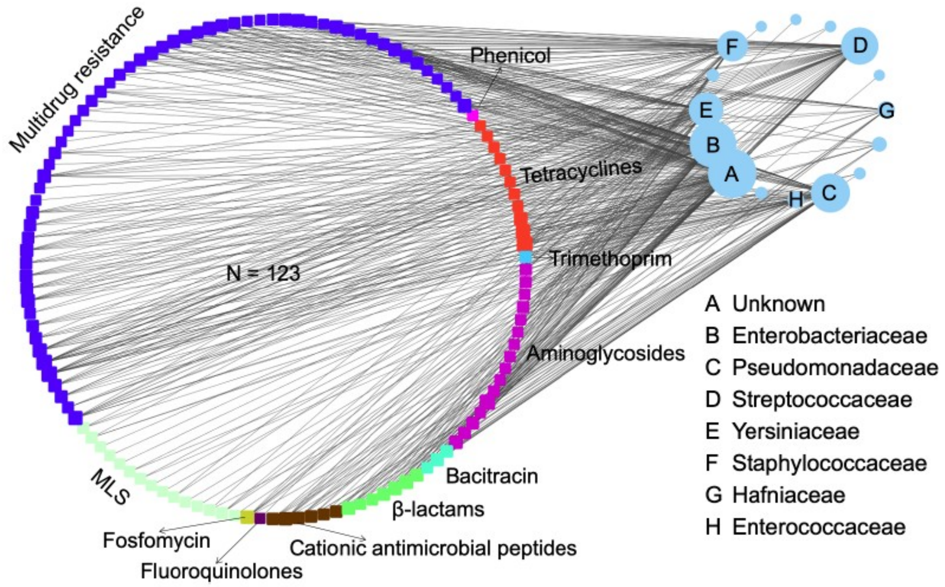

Supplementary Figure 8: The predicted bacterial families of ARGs observed in raw milk from South Carolina. There were 123 ARGs originating from 15 bacterial families were detected in milk samples. Families harboring <10 individual ARGs were not labeled, and detailed information of ARGs and bacteria networks is available in Supplementary Data 2. Edge thickness indicates the normalized abundance of ARGs (rounded squares; colored by class of antibiotics) from a predicted bacterial family (circles). Node size represents the number of connections (degree).
