# Supplementary material for: Reservoirs of antimicrobial resistance genes in retail raw milk
Source: Microbiome. 2020 Jun 26;8:99. doi: 10.1186/s40168-020-00861-6 (PMC7320593; doi:10.1186/s40168-020-00861-6)
Supplement: Supplementary file 14 [file 40168_2020_861_MOESM7_ESM.docx]

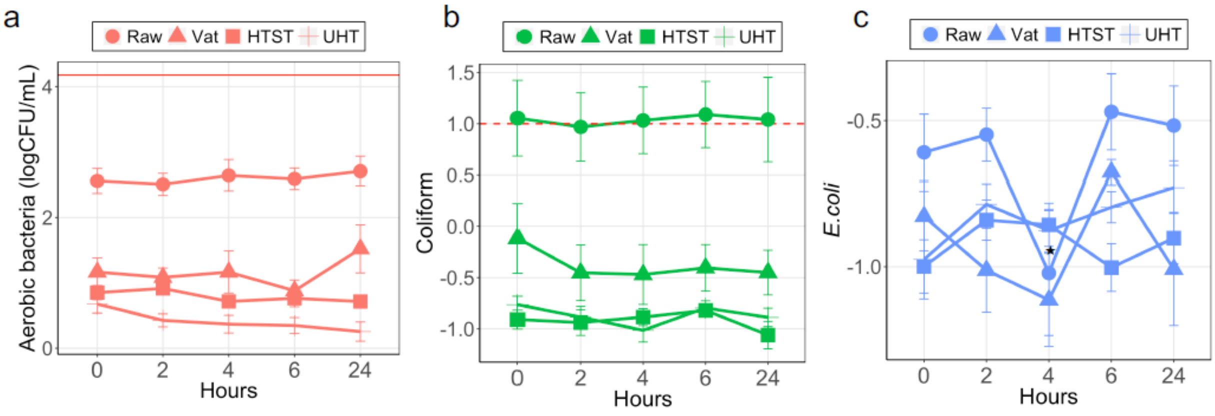

Supplementary Figure 1: The bacterial population in retail milk over incubations at 4°C. **a**, Total aerobic bacteria **b**, Coliform **c**, *E. coli* populations during the 4°C incubation within 24h. A total of 960 milk samples from California were analyzed; Raw (n=240), HTST (n=360), Vat (n=120), and UHT (n=240). Solid and dashed horizontal lines (red) represent the California milk limit for aerobic bacteria (< 15,000 bacteria/mL) and coliform (< 10 coliform/mL), respectively. A linear mixed model (LMM) from the lme4 package (version 1.1.21) in R was implemented to test for associations between the incubation time and the bacterial population in each type of milk. ⋆*P* < 0.05 for comparison with start point (0 hour) in raw milk.
