# Supplementary material for: Reservoirs of antimicrobial resistance genes in retail raw milk
Source: Microbiome. 2020 Jun 26;8:99. doi: 10.1186/s40168-020-00861-6 (PMC7320593; doi:10.1186/s40168-020-00861-6)
Supplement: Supplementary file 15 [file 40168_2020_861_MOESM8_ESM.docx]

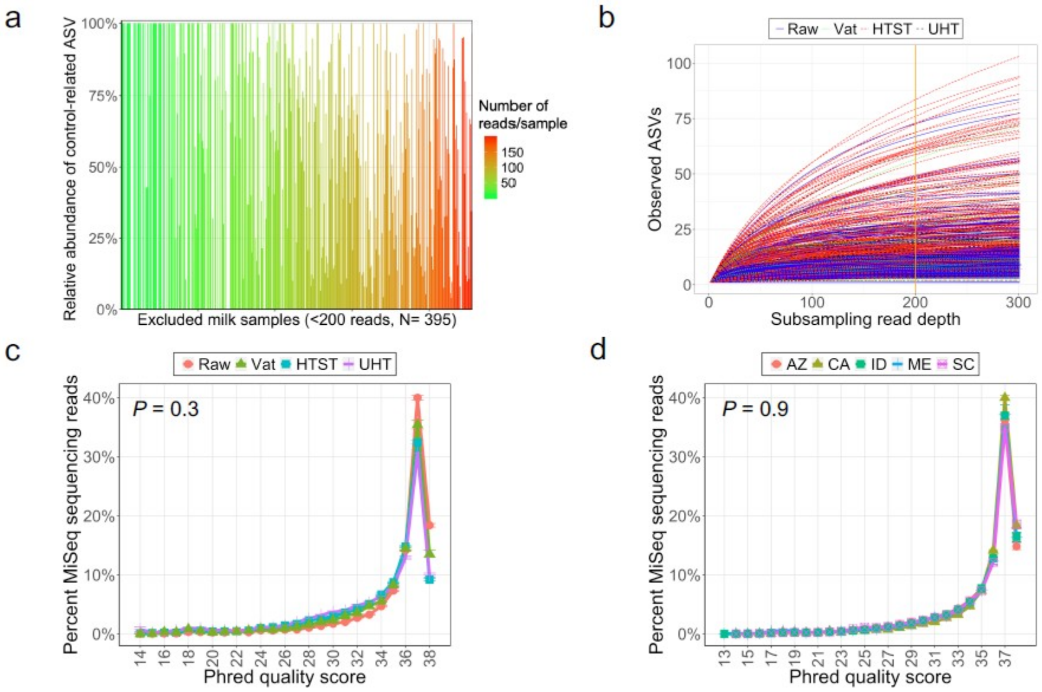

Supplementary Figure 2: The descriptive analysis of 16S rRNA gene sequencing in retail milk. **a**, The relative abundance of 15 shared ASVs (with extraction controls) in excluded low-read milk samples (n=395). Milk samples were ranked by sequencing depth. **b**, The rarefaction curve of observed ASVs across types of retail milk in California. The vertical orange line indicates the subsampling read depth for rarefied feature table. **c**, The distribution of sequencing reads by phred quality score in California retail milk. **d**, The distribution of sequencing reads by phred quality score in raw milk across states in the United States. A Kruskal-Wallis test was used to assess the statistical significance of sequencing quality variance.
