# Supplementary material for: Reservoirs of antimicrobial resistance genes in retail raw milk
Source: Microbiome. 2020 Jun 26;8:99. doi: 10.1186/s40168-020-00861-6 (PMC7320593; doi:10.1186/s40168-020-00861-6)
Supplement: Supplementary file 16 [file 40168_2020_861_MOESM9_ESM.docx]

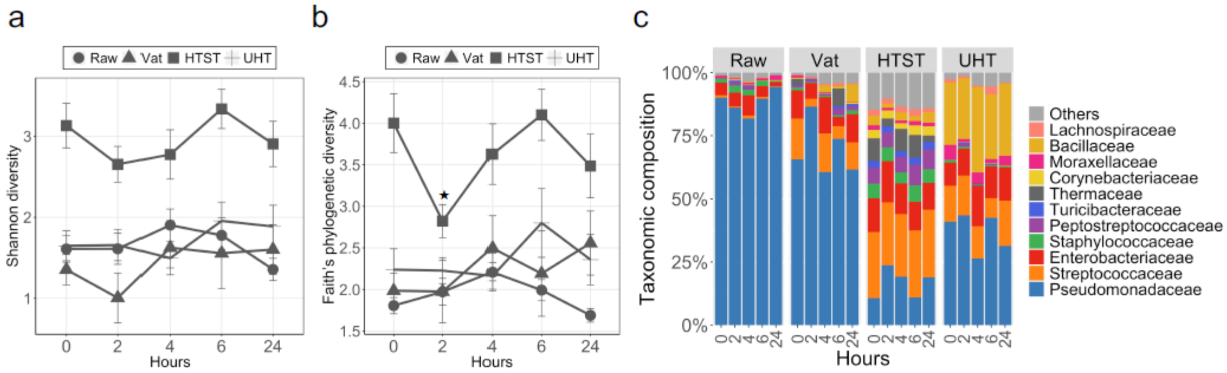

Supplementary Figure 3: The microbiota profiles of retail milk over 4°C incubations. **a**, alpha diversity as measured by Shannon index in retail milk over 4°C incubation. **b**, alpha diversity as measured by Faith's phylogenetic diversity (PD) in retail milk over 4°C incubation. **c**, Bar plot depicting the relative abundance of bacterial families over time; bacterial families which has a relative abundance less than 1% were grouped into "Others". A linear mixed model (LMM) from the lme4 package (version 1.1.21) was implemented to test for associations between the incubation time and the alpha diversity in each type of milk. ⋆$P < 0.05$ for comparison with start point (0 hour) in HTST milk.
